# Environmental Justice and Carbon Pricing: Can They Be Reconciled?

**DOI:** 10.1002/gch2.202200204

**Published:** 2023-02-28

**Authors:** James K. Boyce, Michael Ash, Brent Ranalli

**Affiliations:** ^1^ Political Economy Research Institute University of Massachusetts Amherst 418 N Pleasant St Amherst MA 01002 USA; ^2^ Department of Economics and School of Public Policy University of Massachusetts Amherst 412 N Pleasant St Amherst MA 01002 USA; ^3^ Cadmus Group 410 Totten Pond Road, Suite 400 Waltham MA 02451 USA

**Keywords:** carbon cap, carbon dividends, carbon price, carbon tax, environmental justice

## Abstract

Carbon pricing has been criticized by environmental justice advocates on the grounds that it fails to reduce emissions significantly, fails to reduce the disproportionate impacts of hazardous co‐pollutants on people of color and low‐income communities, hits low‐income households harder than wealthier households, and commodifies nature. Designing carbon pricing policy to address these concerns can yield outcomes that are both more effective and more equitable.

## Graphical abstract

Carbon pricing has been criticized by environmental justice advocates on the grounds that it fails to reduce emissions significantly, fails to reduce the disproportionate impacts of hazardous co‐pollutants on over‐burdened communities, has a regressive distributional impact, and commodifies nature. Carbon pricing policy can be designed to address these concerns, however, yielding outcomes that are more effective as well as more equitable.

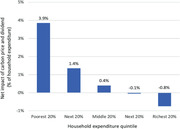

## 1 Introduction

“Carbon pricing” refers to policies that raise the price of fossil fuels by charging money for emitting carbon dioxide into the Earth's atmosphere. This can be done directly by means of a carbon tax (a fixed price per ton of CO_2_) or indirectly by means of a carbon cap (a direct limit on the total amount of CO_2_ that can be emitted, with permits issued up to that limit). In both cases, the simplest and most comprehensive point at which to levy the price is where the fossil fuels first enter the economy—at pipeline terminals, tanker ports, coal mine heads—with firms required to surrender one permit (or pay the tax) per ton of CO_2_ that will be emitted when the fuel is burned. This charge then enters into the prices that are ultimately paid by consumers.

Economists advocate carbon pricing on the grounds that it provides incentives to curb emissions both in the short run (consumers buy less fossil fuel, and less fuel‐intensive goods and services, when their prices rise) and in the long run (incentivizing investments and innovation in energy efficiency and clean energy). As may be expected, powerful opposition to carbon pricing has come from the fossil fuel lobby, which has sought to block legislation or, failing that, to weaken it.^[^ ^1^, ^2^ ^]^ More surprisingly, perhaps, opposition also has come from environmental justice (EJ) advocates. The central goal in EJ is to combat disproportionate environmental harms imposed upon people of color and low‐income communities. Many EJ advocates fear that carbon pricing could exacerbate pollution exposure disparities. This paper focuses on the objections to carbon pricing raised by EJ advocates.

In brief, critics have argued that carbon pricing (i) fails to reduce carbon emissions significantly, (ii) fails to reduce the disproportionate impacts of hazardous co‐pollutants on people of color and low‐income communities, (iii) harms the purchasing power of low‐income households, and (iv) commodifies nature.^[^ ^3^, ^4^ ^]^ Proponents of carbon pricing often, and in our view hastily, have dismissed these criticisms as baseless.

Here, we chart a middle path between dismissal of carbon pricing and dismissal of its critics. The foundation for our position is a basic ethical principle: we believe that the gifts of Nature should be shared in equal measure by all. These gifts include the right to a clean and safe environment—a right recognized in many national constitutions, the most fundamental of legal documents, worldwide^[^ ^5^ ^]^—and the right to share in revenue that is generated by limiting the use of scarce resources. From this perspective, we have a moral imperative both to eliminate the disparate pollution burdens that poison the air and water of overburdened  communities and to halt destabilization of the Earth's climate to protect future generations as well as vulnerable present‐day populations.

Halting the disparate pollution imposed on EJ communities requires, first and foremost, that we take seriously the extent of the problem and recognize the complicity of government policies together with market forces in creating and perpetuating environmental injustice. Solutions require rectifying systemic failures of both the market and the state. EJ and climate stabilization are complementary goals—indeed, climate change itself exacerbates environmental injustice—but we argue here that advancing both goals together requires that explicit EJ provisions be built into the design of climate policy. At a bare minimum, climate policy should guarantee that existing pollution disparities are not exacerbated. Going further, well‐designed design policies can advance the more ambitious goal of reducing environmental disparities.

Halting climate destabilization requires, above all, that we keep fossil fuels in the ground. Carbon dioxide emissions from fossil fuel combustion represent roughly three‐quarters of total greenhouse gas emissions (expressed as CO_2_‐equivalents). To curb these emissions, we must leave fossil carbon where it has lain since before the era of the dinosaurs: buried beneath Earth's surface. Carbon pricing is not the only policy that is needed to keep fossil fuels in the ground, but we argue here that it is an essential part of the climate policy mix.

## 2 Beyond Single‐Policy Politics

**Figure 1:**
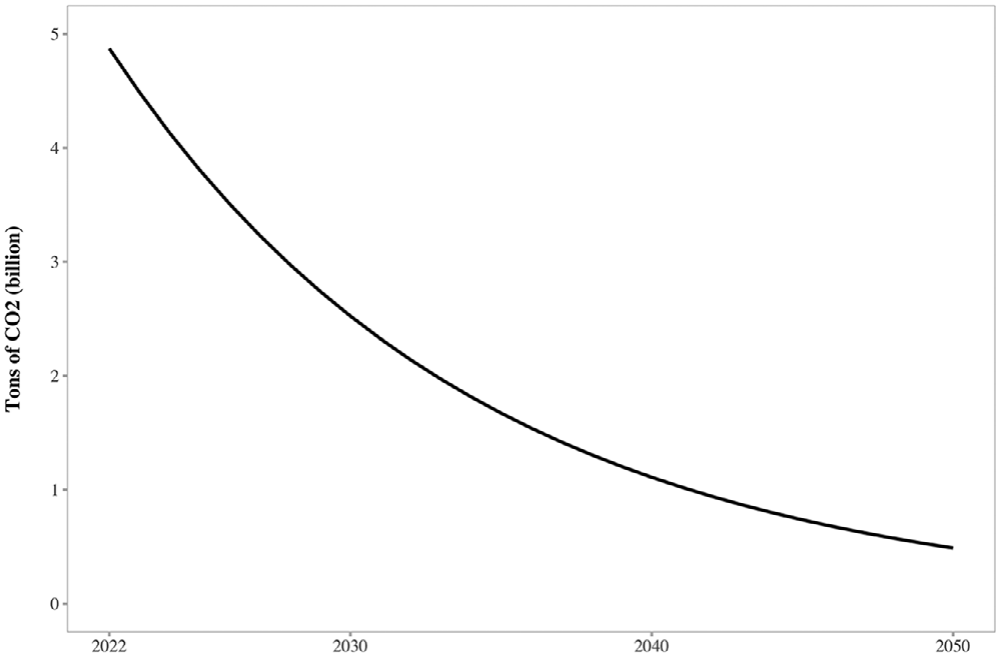
Keeping fossil fuels in the ground. Trajectory of annual global CO_2_ releases (billions of metric tons), 2022–2050, with 8% per year reduction achieving 90% reduction by 2050.

To meet the Paris Agreement's objective of holding average surface temperatures to 1.5–2 °C (3–4 °F) above pre‐industrial levels, the United States and other major consuming countries must cut their emissions to roughly 10% of their current level by the middle of the century. Coupled with measures to sequester atmospheric carbon through improved land management and related practices, this target is consistent with the goal of “net‐zero” carbon emissions by mid‐century.^[^ ^6^, ^7^ ^]^ Cutting emissions by 90% over the next 28 years translates into reductions at the rate of 8% per year (the math is the logic of compound interest operating in reverse), a trajectory shown in **Figure** **1**.

Many policies can help in reaching this goal. Measures to reduce demand for fossil fuels (at any given price) by expanding alternative energy sources and improving energy efficiency play an important role. Carbon pricing typically is introduced alongside such policies, complementing them rather than replacing them.^[^ ^8^, ^9^, ^10^, ^11^ ^]^ California's cap‐and‐trade program, for example, aimed to achieve about 15% of the total emission reductions mandated by the state's Global Warming Solutions Act of 2006, with the remaining 85% coming from other policies to promote clean energy (like renewable portfolio standards for electric power plants) and energy efficiency (like low‐carbon fuel standards for motor vehicles). Carbon pricing does not rule out other climate policies. Neither do other policies rule out carbon pricing.

Climate policy proponents sometimes insist that their own favored policy is the only good one—as if climate policies were mutually exclusive rather than mutually reinforcing. Some have suggested, for example, that carbon prices ought to supplant regulations on CO_2_ emissions,^[^ ^12^ ^]^ while others have argued that regulatory standards and public investment ought to supplant carbon pricing,^[^ ^13^ ^]^ but single‐policy politics is unwarranted in principle and can be counter‐productive in practice, fostering rivalry among potential allies rather than cooperation for the shared goal of protecting the planet.

A similar either–or position embraces the use of “carrots” (like subsidies and tax credits) to reward clean energy and energy efficiency while ruling out “sticks” (like carbon prices) that would penalize fossil fuels. The political logic is that carrots are more palatable to the public.^[^ ^14^ ^]^ Apart from the fiscal issue that these inducements come at a cost to the public purse—carrots do not, as it were, grow on trees—this stance ignores the possibility that revenue from carbon pricing can be recycled directly to the public, as discussed below, effectively turning sticks into carrots for most households, especially those most at risk from fuel price increases.

Judging from past experiences, demand‐side policies by themselves are not likely to curb emissions swiftly and steeply enough to attain the Paris goal for climate stabilization. For example, the landmark climate bill that President Biden signed into law in August 2022, an investment and tax credit package hailed as “the most ambitious climate action undertaken by the United States,” is expected by its proponents to cut emissions 40% below their 2005 level by 2030.^[^ ^15^, ^16^ ^]^ This is equivalent to a 30% reduction below the current level, whereas the Paris‐consistent trajectory shown in Figure 1 would require a reduction of roughly 50%.

For this reason, we also need policies that operate on the supply side to limit directly the total amount of fossil fuels that are burned. One such strategy, widely endorsed by climate justice advocates, is to halt further extraction by blocking new pipelines and new drilling for oil and gas. A more comprehensive variant of this strategy is the call for a “managed decline” of fossil fuel production to be led by the governments of wealthy countries.^[^ ^17^, ^18^, ^19^, ^20^ ^]^ Yet even Norway, where this has been considered more seriously than elsewhere, so far has declined to commit to reducing extraction.^[^ ^21^ ^]^

If efforts to curtail fossil fuel extraction succeed, one consequence of reduced supplies will be upward pressure on fossil fuel prices. In this respect, any policy that restricts supply is a carbon pricing policy. The effect would be comparable to that of OPEC‐led cuts in oil production. If the higher prices lure other suppliers to step up production to fill the resulting breach, dampening or eliminating the price effect, this strategy will fail to protect the climate. If the supply restriction does have a lasting impact on output and emissions, a side effect of the higher prices will be a substantial transfer of wealth from consumers to those producers that continue extracting fossil fuels. Neither outcome can be regarded as a triumph for climate justice.

An alternative supply‐side strategy is to put a hard ceiling on the total amount of fossil carbon allowed to enter the economy—in other words, a cap. One attraction of this strategy is that it can be implemented by any nation, consumer countries (which may have a stronger incentive to act) and producer countries alike. In this respect, a cap is akin to a consumer boycott. The managed decline in fossil fuels here results not from an agreement to limit extraction but from the decision to limit purchases. A carbon cap is regarded as a carbon pricing policy since its effect is similar to that of a carbon tax. A tax raises fuel prices directly; a cap raises them indirectly by limiting supply. In this respect, a cap is akin to a curb on extraction—with the difference that the extra money paid by consumers can be channeled back to the public or to other, more climate‐friendly uses instead of into higher profits for firms that continue to produce fossil fuels.

We advocate carbon pricing via a cap not because it is an “elegant” policy, nor because we regard a carbon price as an end in itself. Rather we do so because we recognize that supply restriction—and the de facto carbon pricing that accompanies it—is a crucial piece of the policy mix required for climate stabilization. If, contrary to past experiences, demand‐side policies were to prove sufficient to achieve emissions reductions at the necessary scale and speed, the supply‐side cap would only provide a backstop, an insurance policy that is never called upon, but if other policies do not prove sufficient, supply‐side restrictions anchored to the required emissions trajectory will be crucial in attaining the climate stabilization goal.

A predictable corollary of any restriction on supply is higher fossil fuel prices. We have crucial choices, however, as to where the money goes. We argue below that the bulk of the revenue from carbon permit auctions (or alternatively from a carbon tax) should be recycled directly to the public as equal per person dividends, as a type of universal income funded by the protection of Earth's climate.

**Figure 2:**
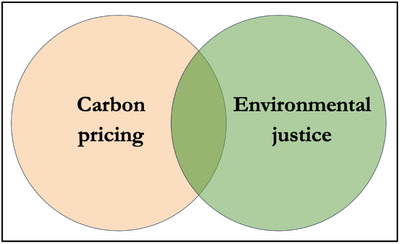
Intersection between carbon pricing and environmental justice.

Notwithstanding their differences, there is a substantial overlap between the goals of decarbonization and environmental justice (**Figure** **2**). This paper sets out four design principles for policies that are compatible with the intersection of the two sets.

## 3 Principle #1: First Quantity, then Price: A Hard Limit on Carbon

Carbon pricing policies today cover more than one‐fifth of fossil fuel emissions worldwide, but they have not had great impact on the growth of emissions, let alone brought about the rapid decreases that are needed to achieve the Paris goal. The main reason is that carbon prices have been set too low. Only four relatively small countries—Sweden, Finland, Switzerland, and Lichtenstein—currently have prices above the range of USD 40–80 per metric ton of CO_2_‐e that many economists consider the minimum needed to begin to make a serious dent in emissions.^[^ ^22^ ^]^

The 40‐to‐80 dollar price range is best seen as a starting point. A helpful rule of thumb is that one dollar per ton of carbon dioxide emissions translates into one cent per gallon of gasoline. A price of USD 40–80 per ton thus would raise gasoline prices at the pump by 40–80 cents. Prices rose by considerably more than this in the first six months of 2022, but few claimed this meant we were securely on the road to resolving the climate crisis.

It is difficult (impossible, really) to predict what carbon price trajectory will be needed to meet the Paris target. The answer depends, among other things, on the full mix of climate policies that are adopted and on how rapidly technological and institutional changes bring down the cost of clean energy alternatives. For example, stricter fuel‐economy standards for automobiles coupled with investments in EV charging stations would reduce demand for gasoline, and thereby lower the carbon price needed to meet the emissions reduction pathway.

Efforts to prescribe the “right” carbon price based on a social cost of carbon (SCC) derived from cost‐benefit analysis are unlikely to ensure a Paris‐consistent emissions reduction trajectory.^[^ ^23^, ^24^ ^]^ When economists estimate the SCC, they take it upon themselves to be the arbiters of the permissible level of global temperature increase. The results can be disconcerting. For example, William Nordhaus concluded in 2017 that the “optimal” mean increase in surface temperatures above pre‐industrial levels at the end of the current century is 3.5 °C, increasing further thereafter.^[^ ^25^, ^26^ ^]^ In recent years, however, some economists have deferred to the 1.5–2 °C limit urged by climate scientists and endorsed in the Paris Agreement, as we do here, advocating target‐consistent carbon pricing.^[^ ^27^, ^28^ ^]^

Uncertainties regarding what other policies will be adopted and how prices will affect fuel demand underscore the importance of a hard limit on total fossil carbon consumption. The number of permits issued would decline over time as depicted in Figure 1. Only fossil fuel firms would be eligible to hold these permits; they would then be required to surrender one permit for each ton of CO_2_ that will be emitted when the fuel they bring into the economy is burned. Implementing this policy at the point where fossil fuels first enter the US economy would involve fewer than 2000 firms nationwide, so the administrative cost would be modest.^[^ ^29^ ^]^ A further advantage of such an “upstream” system—as opposed to a “downstream” one that applies to users of fossil fuels rather than initial suppliers—is that it is comprehensive, covering all fossil fuels regardless of where they are ultimately used.

Rather than allocating permits to corporations free‐of‐charge and then allowing the firms to buy and sell them in a “cap and trade” system, the permits can be auctioned. Permit auctions held at regular intervals (for example, quarterly), coupled with a provision that firms can bank some of their permits for later use, would eliminate any need for permit trading.

In theory, a carbon tax that adjusts over time—the tax rate rising automatically whenever emissions fail to decline enough—could also keep the economy on the required emissions reduction path, but a hard cap with auctioned permits is a more straightforward and proven way to achieve the targeted result. For example, quarterly auctions of carbon permits for power plants have been held since 2009 in the northeastern US states in the Regional Greenhouse Gas Initiative. As carbon tax proponents point out, certainty that the carbon price will rise in future years can help incentivize long‐term investments in energy efficiency and alternative energy. For this reason, the ideal carbon pricing policy would include both a cap on total emissions and a rising floor price—the floor price effectively serving as a baseline carbon tax.^[^ ^30^, ^31^ ^]^

To be certain that the carbon pricing policy achieves the emissions reduction trajectory, it must rule out “offsets.” Offsets allow firms to evade the carbon cap (or tax) by taking steps that ostensibly compensate for their continuing emissions, like planting trees, or refraining from cutting existing forests, or paying others to undertake these activities in a “carbon credit” market. Offsets suffer from the problems of additionality (is the offsetting action genuinely a new reduction in emissions or is it an exercise in labeling for profit?), verifiability (does the action really happen?), and perishability (will the offsetting action endure as long as atmospheric CO_2_?).

Policies to sequester carbon and to reduce greenhouse gas emissions from other sources are certainly needed, but these should be undertaken in addition to cutting fossil fuel emissions, not instead of doing so. Keeping fossil fuels in the ground is not the only thing we must do to address the climate crisis. It is simply the most important.

Past carbon pricing programs have not been terribly effective in reducing the use of fossil fuels, as EJ advocates and other critics have observed.^[^ ^3^, ^4^, ^24^, ^32^ ^]^ The reasons include low carbon tax rates, insufficiently strict caps, incomplete coverage, and loopholes arising from offsets, but this need not be the case. Here, we summarize the basic requirements for an effective carbon pricing policy. First, it includes a hard cap that tightens over time (not simply a price mechanism). Second, it enforces the cap by means of permits that are auctioned at regular intervals to first sellers of carbon fuels (one permit to be surrendered for each ton of CO_2_ that will be released when the fuel is burned), with no permit trading necessary. Third, the policy is free from offsets or other loopholes. Carbon pricing can be implemented alongside other decarbonization policies, but no combination of policies is guaranteed to be effective in ensuring a steep and swift reduction in emissions unless it includes an enforceable mechanism to keep fossil fuels in the ground. The policy outlined above does this.

## 4 Principle #2: Protect the Air: An EJ Mandate for Emissions of Hazardous Co‐Pollutants

Because the climate impacts of carbon dioxide are global, carbon pricing proponents sometimes argue that “carbon is carbon” and insist that it does not matter where emissions reductions occur. This claim ignores the fact that fossil fuel combustion simultaneously releases a host of “co‐pollutants” that impact nearby communities, including particulate matter, sulfur dioxide, nitrogen oxides, and other hazardous air pollutants.^[^ ^33^, ^34^ ^]^

EJ communities are disproportionately affected by pollution, including harmful air pollutants released by fossil fuel combustion.^[^ ^35^, ^36^, ^37^, ^38^, ^39^, ^40^, ^41^ ^]^ As shown in **Table** **1**, the exposure of racial and ethnic minorities (black, Hispanic, Asian‐Pacific Islanders, and Native Americans) and low‐income households to particulate matter emissions from refineries and power plants, for example, is considerably higher than their shares in the total US population.

**Table 1:** Minority and poverty shares of particulate matter exposure from refineries and power plants

	Minority share^a)^	Poverty share
Petroleum refineries	59.5	24.0
Power plants	38.3	15.8
Nationwide population	34.2	13.5

^a)^ Based on emissions weighted by population living within 2.5 miles. Minority share is the share of racial and ethnic minorities in total population. Poverty share is the share of people living below the Federal Poverty Line in total population.^[^ ^33^ ^]^

Policies to address climate change affect the activities and location of much of the polluting part of the economy. This realignment may involve large, one‐time changes with repercussions that last for decades. If the policies governing this transition fail to guarantee tangible environmental gains in EJ communities, they are not likely to win enthusiastic support from EJ advocates, and they will miss an important opportunity to redress longstanding environmental injustices.

The World Health Organization has identified ambient (outdoor) air pollution as a leading cause of premature mortality. A recent *Lancet* study concludes that this pollution is responsible for more than four million deaths each year across the world.^[^ ^42^ ^]^ Fossil fuel combustion is the largest source of this pollution.^[^ ^43^ ^]^ The death toll is especially high in China, India, and other newly industrializing countries, but air pollution causes hundreds of thousands of deaths in high‐income countries, too, including 38 000 per year in the United States according to WHO estimates,^[^ ^44^ ^]^ and possibly more.^[^ ^45^ ^]^

The central objection to carbon pricing voiced by EJ advocates has been that it could widen pollution exposure disparities. Carbon pricing lets polluters decide whether, where, and how to curtail emissions or choose instead to pay the price. Economists hail this flexibility as one of the policy's chief attractions because lower‐cost options for emissions reductions will be preferred. In the absence of constraints, however, the policy's flexibility allows continued or even increased emissions in specific locations, even if total emissions in the covered territory are reduced. For example, if carbon pricing encourages a shift from coal‐fired electricity generation in one location to gas‐fired power generation (which produces less carbon per megawatt hour) in another, emissions in the latter locality will go up. The risk of continued or higher emissions in EJ communities is compounded if the policy allows offsets.

Possible adverse impacts on local air pollution—known as the “hot spot problem” in the environmental economics literature—were the main concerns raised by EJ advocates who opposed the introduction of California's cap‐and‐trade system for carbon emissions a decade ago. At the time, their fears were dismissed by many of the policy's proponents, who assumed that lower carbon emissions would be accompanied by lower co‐pollutant emissions across‐the‐board, despite local variations in the extent of reductions.

Subsequent events have shown that the EJ concerns were well‐founded. Neighborhoods near facilities regulated in California's cap‐and‐trade program that showed the least improvement in greenhouse gas emissions—often experiencing absolute increases—generally had higher‐than‐average percentages of people of color and low‐income households.^[^ ^46^, ^47^, ^48^ ^]^ Similarly, a recent analysis of power plant emissions in the Regional Greenhouse Gas Initiative of the northeastern states found that electricity generation from gas‐fired plants has risen faster in EJ communities.^[^ ^49^ ^]^

**Table** **2** reports the changes at California facilities that experienced large increases in GHG emissions (more than 200 000 metric tons) in the first 5 years of the cap‐and‐trade program (2011/12 to 2016/17) and are located in densely populated areas (with more than 100 000 people living within 5 miles of the facility). The data were obtained from the California Air Resources Board's Pollution Mapping Tool that covers facilities in the state's Mandatory Reporting of Greenhouse Gas Emissions system.^[^ ^46^ ^]^

**Table 2:** California facilities with increased carbon emissions under cap‐and‐trade

Facility	CO_2_‐equivalent emissions change	Demographics within 5‐mile radius		Co‐pollutant emissions change		
		Population	People of color	NOx	SO_2_	PM_2.5_
NRG Energy, El Segundo	119.5%	349 481	61%	28.7%	0.0%	−77.8%
LADPW Scattergood, Playa del Rey	49.2%	336 664	58%	12.0%	−64.3%	−37.4%
Tesoro Los Angeles Refinery, Carson	41.2%	595 242	88%	138.8%	17.0%	50.8%
Chevron Refinery, El Segundo	7.4%	399 940	63%	12.5%	−22.2%	19.8%
Chevron Refinery, Richmond	5.9%	161 146	80%	−14.0%	−3.0%	23.5%

The two facilities that experienced the largest percentage increases in carbon emissions are gas‐fired electricity generation plants; the other three are refineries. The share of people of color in the surrounding population ranges from 58% to 88%. California's relatively stringent air pollution controls led to decreased co‐pollutant emissions in some cases, notwithstanding increased carbon emissions, an outcome that illustrates the potential for regulatory remedies, but all five facilities showed increases in emissions of at least one major co‐pollutant: nitrogen oxides (NOx), sulfur dioxide (SO_2_), or fine particulate matter (PM_2.5_).

At first glance, these findings may appear to contradict those of a study by two researchers at the University of California Santa Barbara that concluded that pollution exposure gaps between EJ communities and others narrowed as a result of the state's cap‐and‐trade program.^[^ ^50^, ^51^ ^]^ The study excluded refineries and electric power plants from the analysis, despite the fact that these accounted for three‐quarters of the emissions regulated under the program, on the grounds that these sectors could have been impacted by other regulatory measures (such as renewable portfolio standards for electricity producers), but other regulatory measures typically are part of the setting within which carbon pricing programs are introduced, as noted above. Moreover, these measures would have contributed to lower emissions, not the opposite; that is, the increases reported in Table 2 occurred despite other policies, not because of them.

Moreover, instead of analyzing the demographic characteristics of communities impacted by facilities where emissions increased, the study applied an estimated “common percentage effect” to assess the impact of cap‐and‐trade on all regulated facilities. Because facilities impacting EJ communities generally had higher emissions than others at the outset, applying this common percentage to them yields larger absolute predicted reductions. In other words, the study's conclusions are based on what the authors consider to be a general rule, rather than spatial variations across locations, but variations are an inherent feature of carbon pricing systems, and it is the places where pollution burdens are not reduced, or even go from bad to worse, that are the focus of EJ concerns.

To ensure that carbon pricing policies do not exacerbate EJ disparities in exposure to localized co‐pollutants, at a minimum they should mandate real‐time agency monitoring of pollution levels in vulnerable communities and provide for corrective measures whenever adverse impacts are found. The State of Washington included such a provision in its 2021 Climate Commitment Act.^[^ ^52^ ^]^

More robustly, an EJ Guarantee could be built into carbon pricing policy by mandating that environmental agencies:

- Use EJ screening tools to identify vulnerable communities where co‐pollution emissions from fossil fuel combustion are responsible for a significant share of environmental health risks.
- Monitor ambient air quality in these communities and co‐pollutant emissions from sources in and near these communities.
- Record and report these data at the level of monitor and pollutant, making this information available to the public via the Internet in real time to help empower communities to participate in the environmental decision‐making.
- Implement measures to ensure that co‐pollutant emissions impacting vulnerable communities are reduced by at least 8% per year, matching the mandated overall carbon emissions reduction.

If concerns about co‐pollutant hot spots turn out to be unwarranted (as some carbon pricing proponents still maintain), then the EJ Guarantee would simply be a precautionary measure with no effect on the policy's outcome, but this is not a sound reason to oppose the guarantee, any more than confidence that one's house will not burn down is a sound reason to forgo fire insurance.

If the EJ Guarantee does, in fact, alter the policy's outcome—so that the spatial pattern of emissions differs from what would have resulted without it—then it will serve as a guardrail for environmental justice. Whatever its effect, the guarantee would not diminish the efficacy of the policy in reducing carbon emissions. Moreover, in straightforward efficiency terms—apart from the justice rationale—the added benefits from improved air quality and public health may outweigh any added costs. For example, a simulation study concluded that incorporating clean air and EJ goals in a 20% decarbonization of the US electric power sector would add no more than 5% to total implementation costs, and that the resulting health benefits would be more than twice the extra cost.^[^ ^41^, ^53^ ^]^ The EJ Guarantee is consistent with recent research finding that a location‐specific approach to racial‐ethnic exposure inequalities is more effective and efficient than conventional regulatory approaches based on sectoral best available control technology (BACT) mandates or regional pollution concentration standards like the National Ambient Air Quality Standards (NAAQS).^[^ ^54^ ^]^

Co‐pollutant impacts are relevant not only to carbon pricing but to many other climate policies as well: for example, the Clean Energy Standards and Renewable Portfolio Standards that mandate a rising share of renewables in electricity generation.^[^ ^41^, ^55^ ^]^ Concerns about co‐pollutant impacts are not an argument against carbon pricing or other climate policies; rather, they are an argument for explicitly building EJ and clean‐air objectives into policy design.

## 5 Principle #3: Protect Household Incomes: Climate Dividends for All

The most politically damaging criticism of carbon pricing—coming from across the political spectrum, not only from EJ advocates—is that higher fuel prices would harm consumers by raising their cost of living. This is the main reason why carbon pricing policies, when they are implemented, usually establish a price too low to have much impact. It also helps explain why carbon pricing has practically disappeared from the US policy debates under the Biden administration.

As a share of household income, the harshest impact of carbon pricing tends to be felt by lower‐income families. Even though they consume much less fossil fuel than richer families in terms of absolute quantities, the relative share of fuel expenditure in their household budgets is often higher, particularly in the industrialized countries.^[^ ^56^, ^57^, ^58^ ^]^ In other words, in the absence of countervailing measures, carbon pricing is regressive, hitting the poor harder than the rich. In a cruel irony, those who bear the greatest harm from climate destabilization and air pollution also bear the greatest burden from increases in the price of fossil fuels.

There is a crucial difference, however, between price increases that boost the profit margins of energy corporations and the price increases that would result from a cap on emissions or a carbon tax: where the money goes. With auctioned permits under a carbon cap (in contrast to OPEC price hikes or permit giveaways under cap‐and‐trade) or with a carbon tax, the extra money paid by consumers becomes government revenue. With a stringent cap or a robust tax, the amount of revenue could be substantial.

If all or most of this revenue is recycled directly and in a timely manner to households on an equal per‐person basis as climate protection dividends (also known as “carbon dividends”), akin to stimulus checks, the impact of carbon pricing on family incomes would be transformed. Instead of a regressive effect, the outcome would be strongly progressive. Most low‐income households would come out well ahead in purely financial terms, receiving more in dividends than they pay in higher fuel costs, without even counting benefits from protecting the environment. The purchasing power of most middle‐class households would be kept whole. High‐income households, because they consume above‐average amounts of carbon (via expenditures on items such as jet travel, outsized homes, yachts and helicopters), would pay more than they receive in dividends—but they can afford it. To ensure both transparency and universal coverage, dividends should be paid via electronic bank transfers (or checks in the mail) rather than returned to the public as an adjustment to income taxes or other government benefits or payments.

**Figure 3:**
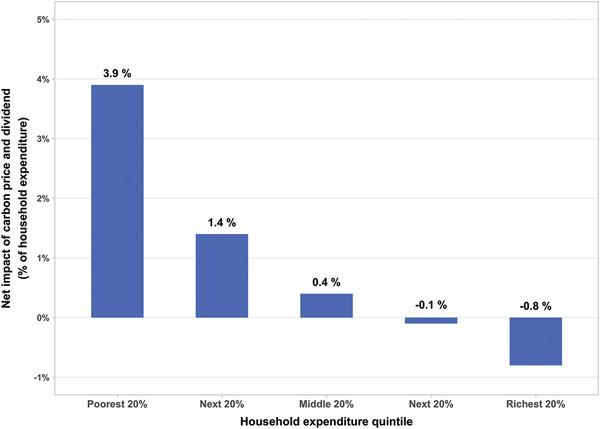
Net effect of $50/ton CO_2_ price coupled with dividends in the United States. Average net impact of carbon price and dividend as a share of average household expenditure by US expenditure quintiles.^[^ ^57^ ^]^

The net distributional impact in the United States, computed on the basis of household expenditure patterns and sectoral input‐output data,^[^ ^57^ ^]^ is shown in **Figure** **3**. At a price of $50 per ton, returning 100% of the carbon revenue as dividends paid equally to all individuals would lift disposable incomes for the poorest 60% of households after paying the higher price for fuel. Only the richest one‐fifth of households would see a noticeable net cost. At the higher prices that would be likely to result from a hard cap tied to a Paris‐consistent trajectory, the distributional pattern would be the same, with the net benefit for working families (and net cost to the most affluent households) being larger.

Apart from dividends one can think of other uses for carbon revenues, some worthy and others not so worthy. High on the list of worthwhile uses are public investments in the clean energy transition and environmental protection, particularly in disadvantaged communities; transitional adjustment assistance for the workers and communities who depended in the past on fossil fuel extraction and processing; and assistance to local governments, including school boards, that also would feel the impact of higher fuel prices. Some lawmakers have proposed dedicating a fraction of the total revenue, say 25%, to these and other uses, with the remainder to be paid to individuals as dividends.^[^ ^59^ ^]^ We fully support public investments for environmental health and equity, but we would prefer to see them funded primarily by progressive taxation with most carbon revenue returned directly to the people.

Some economists have proposed using carbon revenues as a budget‐neutral way to cut income taxes or other taxes. Apart from the fact that carbon revenues often are distributionally regressive (in the absence of dividends), hitting the poor harder than the rich as a percentage of their incomes, such a “green tax shift” would tie government revenue to a source that ultimately diminishes as the clean energy transition is completed.^[^ ^60^ ^]^

Climate dividends paid equally to all would be a type of universal income based on environmental protection. Why pay these dividends to everyone, instead of only to the low‐income households who need them most? In our view, there are compelling reasons for universality, both philosophical and political. From a philosophical standpoint, universal dividends embody the ethical principle that all people own the gifts of Nature—in this case, the right to share in the revenue from use of the limited carbon absorptive capacity of the biosphere—in equal and common measure.^[^ ^59^, ^61^, ^62^, ^63^ ^]^ From a political standpoint, universality helps safeguard the durability of the policy of keeping fossil fuels in the ground throughout the decades needed to complete the clean energy transition, much as universality has protected social security and national health systems.

Environmental justice advocates likewise invoke the ethic of universality when they rebut accusations of NIMBYism (not‐in‐my‐back‐yard insularity) with the reply, “Not in anybody's back yard.” This does not imply the utopian aspiration that pollution will be completely eliminated in the foreseeable future; rather, it means that the burdens of whatever pollution is allowed should not be concentrated in specific communities.

Let us be clear: Climate protection dividends are not a substitute for steps to ensure cleaner air in EJ communities (see Principle #2). Money is not a substitute for a healthy environment or for the power to have a say in environmental outcomes in one's community, but dividends would effectively counter the objection that carbon pricing would hit low‐income households harder than the rich, by reversing the policy's distributional impact.

## 6 Principle #4: Value Nature, Do Not Commodify It

A further objection raised against carbon pricing is that it “commodifies” nature, reducing something that ought to be treated as sacred—the integrity of the planetary ecosystem—into something prosaic, or even profane, that can be bought and sold like soybeans or pork belly futures.^[^ ^64^ ^]^

There is a fundamental difference, however, between valuing nature and turning it into a commodity. When we fail to put a price on carbon and allow emissions free‐of‐charge, we effectively value the resulting climate impacts on present and future generations at zero. This is not treating Nature as sacred; it is treating it as worthless.

Every commodity has a price, but not everything with a price is a commodity. Commodities can be traded, bought and sold repeatedly. Putting a price on emissions need not turn Nature into a commodity, any more than installing parking meters on city streets turns the streets themselves into a commodity. Rather, parking meters charge for use of a scarce resource, helping along with parking regulations to prevent overuse and congestion. A carbon price similarly charges for parking CO_2_ in the atmosphere.

The EJ‐responsive policy we have outlined above—with a hard limit on emissions, safeguards against hot spots, and auctioned permits coupled with dividends—differs markedly from the “carbon markets” established by cap‐and‐trade and carbon credit (aka offset) systems that commodify carbon. Cap‐and‐trade systems often start with free permit giveaways to corporations, allocated according to a formula based on historic emissions. In effect, firms that were responsible for more pollution in the past are rewarded with more permits in the present. The recipients are then free to trade permits with one another—firms that want more permits buying them from those that find it more profitable to cut their own emissions and sell their permits—a feature whose rationale is to allow each firm to decide how many permits they want to use at the prevailing price. If permits are auctioned, rather than allocated free‐of‐charge, no such trading is necessary. If we consider other familiar examples of permits—for parking, driving, hunting, fishing, building, use of landfills, and so on—none of them are tradable. The permit has a price, but it is not a commodity that the holder can resell to others.

Some cap‐and‐trade systems go further, allowing permits to be bought and sold not only by the firms that receive and redeem them but also by financial intermediaries seeking to profit by buying low and selling high. The ultimate source of any such arbitrage profits is the consumer, whose fuel bills now cover the traders’ margins on top of the windfall profits of firms that received free permits. Such full‐blown permit trading creates needless opportunities for market manipulation and speculation. This is not an intrinsic feature of carbon pricing; it is a feature of policies designed to meet the interests of powerful special interests as opposed to consumers.

A further step on the commodification path is taken if carbon pricing systems include offsets that allow firms to continue supplying fossil fuels (or, in the case of downstream systems, burning them) without permits if they pay for something else—like planting trees—that supposedly offsets the emissions for which they are responsible. In this set‐up, those who plant trees get carbon credits that they can sell on the offset market. As we have noted, offsets effectively turn the carbon cap into a sieve. This does not mean that land stewards who take measures to improve carbon sequestration in soils and plant biomass should not be rewarded for this service, but these actions should be undertaken in addition to keeping fossil fuels in the ground, not instead of doing so.

## 7 Concluding Remarks

A carbon price is not an end in itself. Rather, it is a consequence of imposing a binding constraint on the supply of fossil fuels—the price, that is, of a serious commitment to keep fossil fuels in the ground. A mix of policies can be implemented to keep fossil fuels in the ground, not only to reduce demand for them but also to restrict their supply. If demand‐side policies prove sufficient on their own to reduce emissions on a path consistent with the climate stabilization objective ratified by the Paris Agreement, supply restrictions would serve merely as backstop insurance; if demand‐side policies prove insufficient, then the supply restrictions are needed to ensure the necessary emissions reductions. Even the most sanguine of demand‐side policy enthusiasts should not spurn this insurance.

Starting from the ethical premise that the gifts of Nature belong equally to all, this paper seeks to reconcile the twin goals of climate protection and environmental justice. We are convinced there is no intrinsic conflict between them. On the contrary, the two can and should go hand‐in‐hand. Translating this compatibility into practice, however, has proven difficult. Many economists and other proponents of carbon pricing regard it as a vital instrument in the climate policy toolkit, whereas many EJ advocates view the idea with suspicion or downright antipathy. Their reasons for their skepticism cannot be brushed aside lightly, and their fears about being dealt out in the coming energy transition in a replay of past environmental injustices are understandable.

The key to reconciling carbon pricing and environmental justice is to design climate policy with this firmly in mind. To this end, we have outlined four design principles:

- First, to guarantee that carbon pricing is effective in meeting the climate stabilization goal, the policy must be anchored to a hard cap on emissions that declines steadily on a trajectory consistent with net‐zero emissions by mid‐century. Permits to bring fossil carbon into the economy should be auctioned, their number limited by the cap, with a floor price that rises over time. The carbon price that emerges from the cap is not simply a way to curb emissions: it is a result of keeping fossil fuels in the ground by restricting their supply.
- Second, to ensure that carbon pricing reduces disparities in exposure to co‐pollutants from fossil fuel combustion, rather than maintaining or exacerbating these disparities, decarbonization targets should be paired with mandates for improving local air quality in EJ communities overburdened by fossil fuel emissions. These mandates could guarantee, for example, that co‐pollutants will be reduced at a pace that is at least equivalent to the overall reduction in carbon emissions. If co‐pollutant reductions occur simply as a side‐benefit of carbon pricing, then this provision too will serve as backstop insurance; but if not, the EJ guarantee will ensure that environmental injustices are not perpetuated or worsened. Even the most sanguine of carbon pricing proponents should welcome this insurance.
- Third, to counter the regressive impact of carbon pricing on household incomes, most or all of the revenue from permit auctions should be returned directly to the public as equal per‐person dividends. Most households will come out ahead from this carbon price‐and‐dividend policy in straightforward pocketbook terms. Low‐income households generally reap the largest net benefits by virtue of their smaller‐than‐average carbon footprints. Climate protection dividends are a type of universal income derived from charging a price for use of a scarce resource that we own in common, namely the biosphere's limited ability to absorb carbon safely.
- Finally, to guard against the risks that commodification would pose to effectiveness, equity, and public acceptance of carbon pricing, permits should not be tradable, and offsets should be prohibited. Trading is unnecessary if permits are auctioned rather than given away free of charge and would create needless opportunities for market manipulation and speculative activity. Offsets suffer from serious problems of additionality, verifiability, and perishability; they risk turning the cap into a sieve. Measures to sequester atmospheric carbon should be undertaken not as an alternative to keeping fossil fuels in the ground, but instead as another complementary part of the policy mix alongside carbon pricing, regulatory standards, public investment, and transitional adjustment assistance.

In sum, the question is not whether carbon pricing is desirable or not, but whether carbon pricing policies will be designed to be environmentally effective and environmentally just. We believe this is possible.

## Conflict of Interest

The authors declare no conflict of interest.
